# Deep Learning‐Based Prediction Model for Cardiac Resynchronization Therapy Responders Using Electrocardiogram Data

**DOI:** 10.1111/jce.70212

**Published:** 2025-12-02

**Authors:** Hitoshi Mori, Yuya Fujisaki, Syunta Higuchi, Masataka Narita, Daisuke Kawano, Kazuhisa Matsumoto, Wataru Sasaki, Tsukasa Naganuma, Naomichi Tanaka, Kazuhiko Kuinose, Haruka Yamazaki, Hiroki Yamazaki, Wataru Yoshino, Toshiki Takeda, Yoshifumi Ikeda, Ritsushi Kato

**Affiliations:** ^1^ Department of Cardiology Saitama Medical University International Medical Center Hidaka Japan; ^2^ Sera, Inc Meguro Japan; ^3^ Department of Medical Engineer Saitama Medical University International Medical Center Hidaka Japan

**Keywords:** cardiac resynchronization therapy, deep learning, electrocardiogram, responder

## Abstract

**Background:**

Cardiac resynchronization therapy (CRT) is an established treatment for advanced heart failure, but approximately 30% of patients fail to respond. This study aimed to develop and evaluate deep learning models using preimplantation electrocardiogram (ECG) data to predict CRT response.

**Methods:**

We conducted a retrospective analysis of 285 patients who underwent CRT implantations and completed a 6‐month follow‐up. Responders were defined as those exhibiting ≥ 15% left ventricular end‐systolic volume reduction. Three models were developed: ResNet‐18 model trained on ECG images, self‐supervised learning (SSL) enhanced ResNet‐18 model, and LightGBM model trained on time‐series ECG data. Model performance was evaluated using accuracy, positive predictive value (PPV), and negative predictive value (NPV), averaged across 10 random seeds. Model interpretability using Gradient‐weighted Class Activation Mapping (Grad‐CAM) was performed on 36 responder cases.

**Results:**

The SSL + ResNet‐18 model demonstrated the most stable performance (accuracy 78.5% ± 5.5%) and PPV of 81.3%. The ResNet‐18 model achieved the highest PPV of 84.2% but had lower accuracy (74.1%) and larger variability. The LightGBM model exhibited the highest accuracy (79.4%) but the lowest PPV at 72.8%. Grad‐CAM showed that precordial leads were highlighted in 13 cases (36.1%), limb leads in 16 (44.4%), and both regions in 7 (19.4%), indicating heterogeneity in the model's focus and potential diversity in the electrical features contributing to CRT response prediction.

**Conclusion:**

AI models using preimplantation ECG data, particularly those based on image inputs, can effectively predict CRT responders. This approach may enhance patient selection and support personalized therapy strategies in CRT management.

## 1 Background

Cardiac resynchronization therapy (CRT) is a therapeutic strategy for patients with severe heart failure (HF), aimed at improving cardiac function, reducing HF hospitalizations, and decreasing mortality by enabling synchronized left ventricular contraction through pacing [1, 2, 3, 4]. Current clinical guidelines classify CRT implantation eligibility based on the QRS morphology and QRS duration, and these guidelines are widely utilized in clinical practice [5]. Although approximately 70% of patients respond to CRT (responders) with improvements in the left ventricular ejection fraction (LVEF), around 30% do not exhibit LVEF recovery (nonresponders) [6, 7, 8]. For nonresponders, early consideration of intensified pharmacological therapy or transition to a left ventricular assist device (LVAD) is crucial. Preimplantation mechanical dyssynchrony of the left ventricle (LV) is known to correlate with CRT responsiveness [9]. Echocardiography has traditionally been employed to assess dyssynchrony and predict CRT outcomes. The electrocardiogram (ECG), which reflects electrical activity through the QRS polarity and width, also provides insight into cardiac electrical dyssynchrony [8, 10].

Recent advancements in artificial intelligence (AI) have demonstrated significant potential in the medical field, including the analysis of ECG data. Although there have been a few reports on the use of AI for arrhythmia detection in ECG analysis, studies focusing on AI‐based learning for CRT response prediction using ECG data remain largely unexplored [11, 12]. This study aimed to develop a predictive model for CRT responders using preimplantation ECG data, leveraging AI technologies to enhance the precision of patient selection and optimize therapeutic outcomes.

## 2 Methods

### 2.1 Study Population

**Figure 1:**
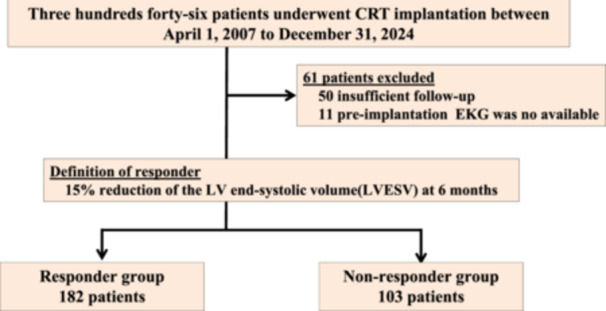
Flow diagram of the patient eligibility. A total of 346 patients who underwent CRT implantations were enrolled in this study. A total of 50 patients were excluded due to insufficient follow‐up, which precluded classification into responder or nonresponder groups. An additional 11 patients were excluded because their preimplantation electrocardiograms were not available for analysis. After those exclusions, 285 patients were included in the final analysis.

The study was a single‐center, retrospective analysis. A total of 346 patients who underwent CRT implantations between April 1, 2007, and December 31, 2024, were enrolled in this study. All patients met the clinical guidelines for a CRT indication (Figure 1). After the CRT implantation, CRT optimization was performed during the follow‐up at least once. A responder was defined as a 15% reduction in the LV end‐systolic volume (LVESV) at 6 months after the implantation (responder group) [13]. Patients who failed to achieve the defined improvement in the LVESV were classified as the nonresponder group. Those who were unable to complete the 6‐month follow‐up were excluded from the analysis. After excluding those patients, the preimplantation ECG data of both the responder and nonresponder groups were used for learning.

This study was conducted in accordance with the provisions of the Declaration of Helsinki and local regulations.

### 2.2 ECG and Learning Models

In recent years, various AI models based on the ECG have been developed. However, there is currently no standardized format for ECG input data or preprocessing protocols. The ECG data used in these studies are collected from a wide range of devices, and preprocessing methods vary significantly between studies. Therefore, it is essential to establish unified standards across the industry and build a robust platform for implementation [14]. In real‐world clinical settings, ECGs are frequently acquired as image data, and this platform conversion presents a practical challenge for model deployment. Therefore, in the present study, we developed both an image‐based prediction model and time‐series‐based prediction model for ECG‐related disease prediction, and compared their respective prediction accuracies.

The predictive models were developed using preimplantation ECG images from both the responder and nonresponder groups. Cases with significant baseline drift or high‐frequency noise were excluded if deemed unsuitable for modeling by electrophysiology (EP) specialists based on visual inspection. ECG image data were exported in a JPEG format, while time‐series data were extracted at 1‐ms intervals in microvolt units and used for model development.

In this study, we developed three prediction models: ResNet‐18 model trained on ECG image data, self‐supervised learning (SSL)–based ResNet‐18 model also trained on ECG image data, and LightGBM model trained on time‐series ECG data. For each model, the data set was split into training and validation sets in an 80:20 ratio at the patient level, ensuring that all ECG images from the same patient were assigned exclusively to either the training or validation set to prevent data leakage. Experiments were conducted using 10 different random seeds, and the average classification performance was calculated. The evaluation metrics included accuracy, positive predictive value (PPV), and negative predictive value (NPV).

### 2.3 Data Preprocessing

#### 2.3.1 Image Data

Raw 12‑lead ECGs were exported as JPEG files. All patient identifiers, labels, dates, and institutional markings were completely erased from the images to prevent the model from learning nonphysiological features. The deidentified images were then resized to 224 × 224 pixels.

#### 2.3.2 Time‑Series Data

For each of the 12 standard leads, we computed five statistical features—mean, standard deviation, minimum, maximum, and median—and concatenated them into a 60‑dimensional feature vector.

## 3 Models

### 3.1 ResNet‑18

Given the successful application of ResNet architectures to ECG image classification in prior work [15], we employed ResNet‐18 from PyTorch initialized with ImageNet pretrained weights (IMAGENET1K_V1) and fine‐tuned on ECG images.

### 3.2 SSL + ResNet‑18

This model employs a two‑stage training strategy designed to leverage large quantities of unlabeled ECG images for more robust feature learning. We implemented the SSL training using the Lightly framework with PyTorch ResNet‐18 architecture [16].

**Figure 2:**
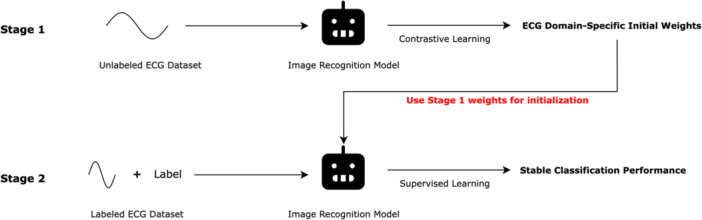
Overall Training Process. The SSL + ResNet‐18 model employs a two‐stage training strategy. In Stage 1, self‐supervised pretraining using SimCLR‐style contrastive learning is performed on ~4000 unlabeled ECG images, where the network learns to pull together augmented views of the same ECG while pushing apart different samples, resulting in robust feature representations invariant to noise and patient variability. In Stage 2, the pretrained ResNet‐18 is fine‐tuned on labeled CRT response data using standard supervised learning. This approach reduces reliance on scarce labeled data, mitigates overfitting, and enhances generalization compared to end‐to‐end supervised training.

(1) Self‑supervised pretraining (SimCLR‑style contrastive objective) on ~4000 unlabeled ECG images encourages the network to learn invariances to noise, orientation, and patient‑specific variability [17]. By pulling together differently augmented views of the same ECG and pushing apart other samples, the model discovers representations that capture subtle morphological patterns related to CRT response (Figure 2).

(2) Fine‑tuning then initializes ResNet‑18 with these pretrained weights and applies the same image augmentations as the baseline model.

This two‑stage approach reduces reliance on scarce labeled responder data, mitigates overfitting, and enhances the stability of downstream classification—yielding feature embeddings that generalize better across random seeds and imaging conditions.

### 3.3 LightGBM

Gradient‑boosted decision tree was trained on the 60‑dimensional statistical feature vectors.

### 3.4 Training Configuration

Additional training parameters are shown in Supporting Information S1: Tables S1–S3.

### 3.5 Evaluation Metrics

We report accuracy, PPV, and NPV, each averaged over 10 random seeds. Standard deviations across seeds assess model stability.

### 3.6 Evaluation of Model Explainability Using Grad‐CAM

**Figure 3:**
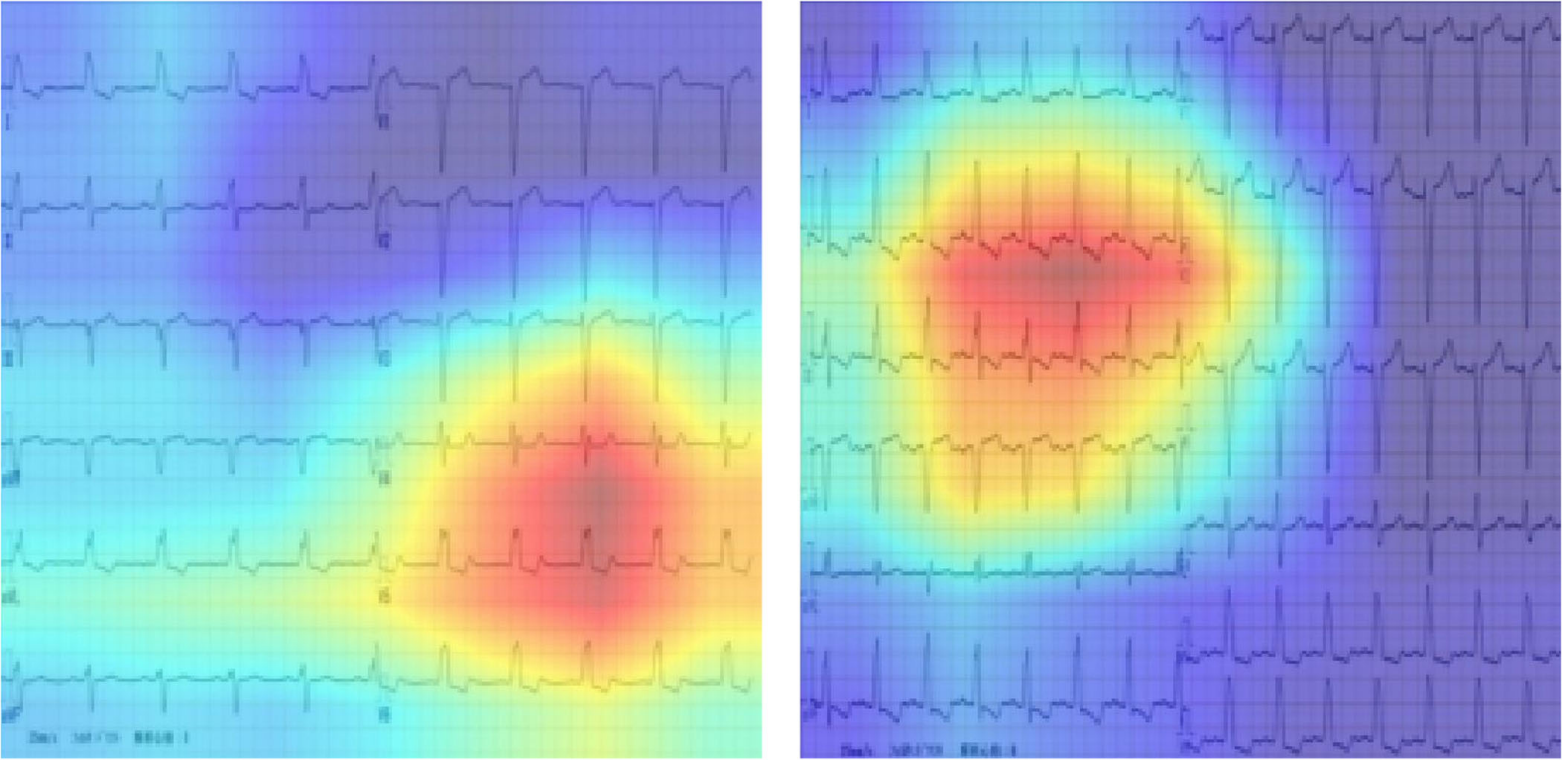
Evaluation of model explainability using Grad‐CAM. Representative examples of Grad‐CAM visualizations in ECGs classified as responders. The left panel shows an ECG with complete left bundle branch block, where the highlighted regions are located in the precordial leads. This suggests that the model identified electrical dyssynchrony in the precordial leads as a key feature associated with a positive CRT response. In contrast, the right panel also shows a responder with complete LBBB; however, the highlighted regions are predominantly in the limb leads.

To assess the explainability of the responder prediction model, Gradient‐weighted Class Activation Mapping (Grad‐CAM) was applied to visualize the regions of the ECG image that contributed most to the model's predictions. This method highlights the areas the model considered most relevant when classifying a case as a responder. Figure 3 presents representative examples of Grad‐CAM visualizations in ECGs classified as responders. The left panel shows an ECG with complete left bundle branch block (LBBB), where the highlighted regions are located in the precordial leads. This suggests that the model identified electrical dyssynchrony in the precordial leads as a key feature associated with a positive CRT response. In contrast, the right panel also shows a responder with complete LBBB; however, the highlighted regions are predominantly in the limb leads. This discrepancy may indicate a case where the model's attention does not align with clinically expected features, suggesting variability in the model's interpretability across individual cases. Grad‐CAM analysis was performed on 36 cases selected from the responder group to evaluate the explainability of the prediction model. Specifically, we examined the proportion of cases in which the highlighted regions were located in the precordial leads.

### 3.7 Statistical Analysis

Continuous variables for which normality is maintained were subjected to the Student's *t* test. To evaluate the classification performance of each model, receiver operating characteristic (ROC) analysis was performed. ROC curves were generated for ResNet18, SSL‐ResNet18, and LightGBM　models, and the area under the curve (AUC) was calculated for each. The comparison of ROC curves was used to assess the discriminative ability of the models. A value of *p* < 0.05 was considered statistically significant unless specified otherwise.

## 4 Results

### 4.1 Patient Characteristics

A total of 50 patients were excluded from the study due to insufficient follow‐up, which precluded classification into responder or nonresponder groups. An additional 11 patients were excluded because their preimplantation electrocardiograms were not available for analysis. After these exclusions, 285 patients were included in the final analysis. Table 1 presents the baseline characteristics and changes in echocardiographic findings.

**Table 1:** Baseline characteristics and the changes in the echocardiographic findings.

	Nonresponder group (*n* = 103)	Responder group (*n* = 182)	*p* value
Patients background			
Age (years)	70 [62–76]	68 [61–75]	0.18
Gender, men, *n* (%)	74 (71.8)	124 (68.1)	0.51
ICM, *n* (%)	22 (21.4)	40 (22.0)	0.90
CLBBB, *n* (%)	19 (18.4)	86 (47.3)	< 0.001
Upgrade, *n* (%)	25 (24.3)	30 (16.4)	0.11
CRTD, *n* (%)	77 (74.8)	147 (80.1)	0.23
AF, *n* (%)	18 (17.5)	24 (13.2)	0.33
Echocardiographic finding			
LVEF (preimplantation), (%)	27.0 [21.3–34.0]	25.0 [20.0–33.0]	0.052
LVESV (preimplantation), (ml)	108.0 [78.6–157.8]	124.4 [91.7–170.4]	0.011
LVEF (postimplantation), (%)	26.0 [20.0–34.0]	39.5 [28.0–47.8]	< 0.001*
LVESV (postimplantation), (ml)	126.0 [92.2–164.0]	68.7 [46.2–112.8]	< 0.001*
LVESV changes, (%)	−10.5 [−27.1 to 4.1]	37.0 [24.9–57.0]	< 0.001*

*Note:* The continuous variables are shown as the median (IQR), and the categorical variables as the number (%).

Abbreviations: AF, atrial fibrillation; CLBBB, complete left bundle branch block; CRTD, cardiac resynchronization therapy with defibrillator; ICM, ischemic cardiomyopathy; LVEF, left ventricular ejection fraction; LVESV, left ventricular end‐systolic volume.

*p < 0.05.

### 4.2 Comparison of Predictive Performance Across Models

We compared the predictive performance of the three models developed in this study: the ResNet‐18 model trained on ECG images, SSL‐enhanced ResNet‐18 model, and LightGBM model based on time‐series data. Performance was evaluated using accuracy, PPV, and NPV, averaged across 10 different random seeds. This comparative analysis allowed us to assess the strengths and limitations of each modeling approach in predicting CRT responders (Table 2, Supporting Information S1: Table S4).

**Table 2:** Baseline characteristics and the changes in the echocardiographic findings.

	Accuracy	Positive predictive value	Negative predictive value
ResNet‐18 model (%)	56.5 ± 12.8	81.8 ± 11.1	49.9 ± 13.0
SSL + ResNet‐18 model (%)	62.1 ± 5.4	78.5 ± 5.5	49.8 ± 5.3
LightGBM model (%)	64.7 ± 5.6	71.2 ± 3.9	50.0 ± 9.5

The ResNet‐18 model had the highest PPV, but it also exhibited the largest standard deviation and lowest overall accuracy among the models. In contrast, the LightGBM model demonstrated the highest accuracy; however, it had the lowest PPV. The SSL + ResNet‐18 model achieved the most consistent performance, with the smallest standard deviation across all evaluation metrics. While the NPV was approximately 50% across all models, the SSL + ResNet‐18 model had the smallest standard deviation for NPV as well, indicating greater stability.

### 4.3 Model Performance

**Figure 4:**
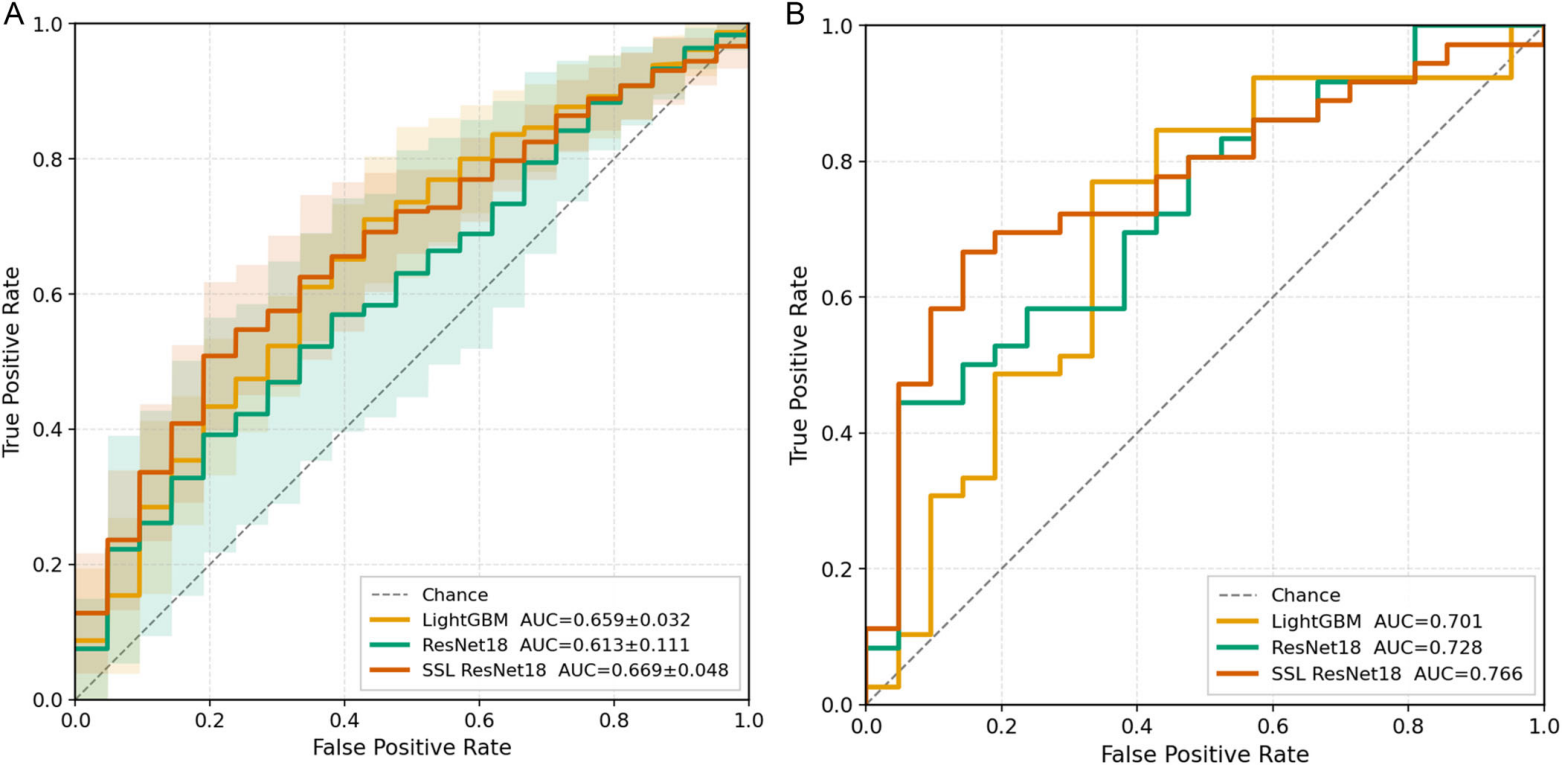
Evaluation of model explainability using Grad‐CAM. (A) Averaged ROC curves obtained from 10 independent training runs with different random seeds. The mean AUCs were 0.659 ± 0.032 for LightGBM, 0.613 ± 0.111 for ResNet18, and 0.669 ± 0.048 for SSL‐ResNet18, with the highest mean AUC observed in the SSL‐ResNet18 model. (B) ROC curves obtained from the best‐performing seed for each model. The AUCs were 0.701 for LightGBM, 0.728 for ResNet18, and 0.766 for SSL‐ResNet18, again demonstrating the best discriminative performance with the SSL‐ResNet18 model.

Figure 4 shows the ROC curves for the different training models. Figure 4A presents the averaged ROC curves obtained from 10 independent training runs with different random seeds. The mean AUCs were 0.659 ± 0.032 for LightGBM, 0.613 ± 0.111 for ResNet18, and 0.669 ± 0.048 for SSL‐ResNet18, with the highest AUC observed in the SSL‐ResNet18 model. Figure 4B illustrates the ROC curves at the best‐performing seed for each model. The AUCs were 0.701 for LightGBM, 0.728 for ResNet18, and 0.766 for SSL‐ResNet18, again showing the best performance with the SSL‐ResNet18 model.

### 4.4 Evaluation of Model Explainability Using Grad‐CAM

Among the 36 responder cases analyzed, Grad‐CAM highlighted the precordial leads in 13 cases (36.1%), limb leads in 16 cases (44.4%), and both the precordial and limb leads in 7 cases (19.4%).

## 5 Discussion

### 5.1 Major Findings

The major findings of our study were as follows:

(1) The SSL + ResNet‐18 model demonstrated the most stable predictive performance, exhibiting the smallest standard deviation across all evaluation metrics (78.5 ± 5.5%). It also achieved the highest AUC among all models, both in the mean across all random seeds and in the best‐performing seed. (2) In 55.6% of the cases, the model focused on the precordial leads when predicting responder status, suggesting attention to features related to ventricular desynchrony. However, in the remaining 44.4% of cases, the model based its prediction solely on the limb leads, indicating variability in the regions contributing to the model's decision‐making. (3) To our knowledge, this study was the first to directly compare time‐series and image‐based ECG representations under identical conditions in CRT prediction, demonstrating the practical utility of self‐supervised learning even with limited unlabeled data, and introducing a novel JPEG‐based workflow that simplifies clinical application.

### 5.2 Utility of the Prediction for CRT Responder

CRT is one of the key treatment strategies for patients with advanced heart failure. However, when the desired therapeutic effect is not achieved, early consideration of alternative treatment options becomes essential. The model developed in this study demonstrated a high PPV in identifying CRT responders. Since responders are more likely to experience improvements in cardiac function and hemodynamic status, early identification of such patients may allow for timely therapeutic optimization [18]. In cases predicted to be responders by this model, intensification of pharmacological therapy and other supportive interventions may be particularly beneficial. On the other hand, in cases not predicted to be responders, optimization of CRT, which is a common cause of nonresponse, may play a crucial role in guiding subsequent treatment strategies [19]. Furthermore, in cases predicted to be nonresponders before implantation, selecting a treatment strategy such as LOT‐CRT from the outset—with the aim of achieving greater ventricular synchrony—may also be considered as a potential therapeutic suggestion [20].

### 5.3 Comparison of Input Representations (Time‑Series vs. Images)

While deep‐learning applications to ECGs have been demonstrated both with signal‑processing + 1D‑convolutional approaches and with STFT‑converted ECG images processed by ResNet‑based models [21, 22], few studies have directly compared these two representations under identical conditions in a CRT patient cohort. In this study, we performed parallel experiments on the same group of CRT patients to elucidate the performance differences and complementarities of each input representation, thereby providing guidance for optimal representation selection in CRT prediction model design.

### 5.4 Effect of Self‑Supervised Learning (SSL) Pretraining

In general domains, SSL methods such as SimCLR applied to approximately 54 566 12‑lead ECGs have been reported to yield around a 1% AUC improvement and enhanced robustness over purely supervised training [23]. Here, using ~4000 unlabeled ECG images from the ECG Images Dataset of Cardiac Patients and a contrastive learning procedure based on SimCLR [17], we demonstrated that SSL can significantly reduce performance variance compared to an ImageNet‑initialized model. This finding suggested that the benefits of SSL could be realized with far less data than previously thought.

Furthermore, we proposed a novel workflow that directly imports paper‑based ECGs as JPEGs—omitting any preprocessing—and showed that this approach greatly enhances clinical practicality compared to the STFT‑based model of He et al. Taken together, this study offers both novel insights and practical engineering guidelines for the design of deep‐learning models to predict CRT responders.

### 5.5 Prediction Model Based on the ECG Image

In recent years, a variety of predictive models utilizing AI technology have been developed in the field of electrocardiography [24, 25]. These models have improved the predictive performance by standardizing the nature of input data, such as digital signal formats. However, a major challenge remains: applying these models to external data requires a unified data platform, which limits their practical applicability. Therefore, standardization of electrocardiographic data is essential [14]. In the present study, we developed a model using ECG image data, which offers enhanced versatility across different clinical environments. The findings suggest that image‐based models may achieve predictive performance comparable to those trained on digital waveform data. This indicates the potential for developing broadly applicable AI models in the future by training on ECG images, thus addressing the limitations posed by data platform discrepancies.

Furthermore, using image‐based ECG data enabled the application of Grad‐CAM analysis, which improved the model's explainability by visualizing the ECG regions that contributed most to the prediction. Importantly, AI‐based prediction models should serve as a clinical decision‐support tool rather than replacing physician judgment. By presenting the reasoning behind predictions in an interpretable visual form, Grad‐CAM may help physicians understand and evaluate the model's output, facilitating informed decision‐making in the management of patients undergoing CRT.

Recently, ultrahigh‐frequency ECG (UHF‐ECG) analysis has been introduced as a novel approach to quantify electrical dyssynchrony and predict CRT response [26, 27, 28]. Similar to the principles underlying UHF‐ECG, image‐based modeling may also capture subtle spatial and temporal variations within the QRS complex that reflect electrical activation heterogeneity. Although our current model used ECG images resized to 224 × 224 pixels for training, future studies employing higher‐resolution image data may allow for more detailed analysis of fine‐grained QRS components, potentially improving the precision of responder prediction.

### 5.6 Limitations

Our study had several limitations. Firstly, CRT responders were defined as those showing a ≥ 15% reduction in LVESV at 6 months postimplantation. However, multiple definitions of response exist, and predictive performance may vary accordingly. In addition, responder status was evaluated only at a single time point, although cardiac function changes dynamically with disease progression and treatment response. Future studies assessing dynamic indicators, such as temporal changes in cardiac function, may better identify hyper‐responders. Secondly, our responder group included patients who underwent CRT upgrades from secondary pacemaker implantations. As a result, ECGs reflecting paced rhythms were also incorporated into the model training. If the model was limited to de novo CRT implantations, the predictive performance might have differed. Third, this study analyzed implantation cases over a 17‐year period, during which significant advancements beyond biventricular pacing occurred, including the introduction of quadripolar leads, the advent of multipoint pacing, and changes in pharmacological therapy. These factors may have influenced the analysis. Finally, the number of ECGs used in this study was relatively small (*n* = 285). To improve model robustness and generalizability, validation using a larger data set is necessary. In addition, due to the limited sample size, there is a potential risk of overfitting, highlighting the need for external validation using independent datasets.

## 6 Conclusion

The prediction model using ECG image data demonstrated performance comparable to that of models based on digital waveform data in identifying CRT responders. The ability to predict responders may provide valuable guidance in determining therapeutic strategies both before and after device implantations.

## Ethics Statement

The study protocol was approved by the hospital's institutional review board (IRB number: 2024‐157).

## Conflicts of Interest

H.M. received lecture fees from Biosense Webster Japan and Boston Scientific Japan. Our department received grant support from Boston Scientific Japan and Abbott Medical Japan.

## Supporting information


**Supporting Table 1:** Self‐Supervised Learning Pretraining Parameters. **Supporting Table 2:** ResNet‐18 Training Parameters. **Supporting Table 3:** Light GBM Parameters. **Supporting Table 4:** PPV and NPV for Each Model – All Seed Results.

## Data Availability

Available upon request.
