# Supplementary material for: Deep Learning‐Based Prediction Model for Cardiac Resynchronization Therapy Responders Using Electrocardiogram Data
Source: J Cardiovasc Electrophysiol. 2025 Dec 2;37(1):157–64. doi: 10.1111/jce.70212 (PMC12794793; doi:10.1111/jce.70212)
Supplement: Supplementary file 1 — Supporting Table 1: Self‐Supervised Learning Pretraining Parameters. Supporting Table 2: ResNet‐18 Training Parameters. Supporting Table 3: Light GBM Parameters. Supporting Table 4: PPV and NPV for Each Model – All Seed Results. [file JCE-37-157-s001.docx]

| **Parameter** | **Value** |
| --- | --- |
| **Hardware** | NVIDIA A100 GPU 40 GB |
| **Training epochs** | 10 |
| **Optimizer** | SGD |
| **LR scheduler** | CosineAnnealingLR |
| **Architecture** | Input: 512, Hidden: 512, Output: 128 |
| **Batch size** | 256 |
| **Color jitter probability** | 0.8 |
| **Random grayscale** | 0.2 |
| **Dropout rate** | 0.5 |
| **Random seeds** | 10 |

**Supplemental table 1: Self-Supervised Learning Pretraining Parameters**

| **Parameter** | **Value** |
| --- | --- |
| **Hardware** | NVIDIA Tesla T4 GPU |
| **Image size** | 224×224 |
| **Learning rate** | 1×10⁻⁴ |
| **Batch size** | 16 |
| **Training epochs** | 30 |
| **Model selection** | Best PPV/NPV balance after epoch 15 |
| **Optimizer** | AdamW |
| **LR scheduler** | CosineAnnealingLR |
| **Image augmentations** | Resize, Rotation ±15°, Color Jitter, Gaussian Blur, Random Erasing |
| **Random seeds** | 10 |
| **Data split** | 80:20 |

**Supplemental table 2: ResNet-18 Training Parameters**

| **Parameter** | **Value** |
| --- | --- |
| **Random seeds** | 10 |
| **Data split** | 80:20 |

**Supplemental table 3: Light GBM Parameters**

|  | | Seed 0 | Seed 2 | Seed 4 | Seed 6 | Seed 8 | Seed 10 | Seed 12 | Seed 14 | Seed 16 | Seed 18 | Average |
| --- | --- | --- | --- | --- | --- | --- | --- | --- | --- | --- | --- | --- |
| ResNet-18 model  (%) | PPV | 90.5 | 73.9 | 80.0 | 85.7 | 100 | 76.9 | 71.4 | 71.4 | 68.3 | 100 | 81.8 |
|  | NPV | 52.8 | 81.8 | 38.5 | 40.0 | 39.6 | 66.7 | 39.5 | 50.0 | 50.0 | 39.6 | 49.9 |
| SSL + ResNet-18 model (%) | PPV | 81.8 | 74.1 | 85.0 | 70.4 | 80.0 | 76.2 | 83.3 | 78.6 | 70.7 | 85.0 | 78.5 |
|  | NPV | 62.5 | 46.7 | 48.7 | 43.3 | 50.0 | 44.4 | 46.2 | 51.7 | 56.3 | 48.7 | 49.8 |
| Light GBM model (%) | PPV | 77.8 | 71.4 | 75.0 | 70.7 | 66.7 | 69.2 | 68.2 | 75.6 | 71.7 | 65.9 | 71.2 |
|  | NPV | 54.2 | 50.0 | 62.5 | 47.4 | 38.1 | 42.9 | 43.8 | 66.7 | 57.1 | 37.5 | 50.0 |

**Supplemental table 4: PPV and NPV for Each Model – All Seed Results**
